# The mitochondrial division inhibitor Mdivi-1 rescues mammalian neurons from anesthetic-induced cytotoxicity

**DOI:** 10.1186/s13041-016-0210-x

**Published:** 2016-03-24

**Authors:** Fenglian Xu, Ryden Armstrong, Daniela Urrego, Munir Qazzaz, Mario Pehar, J. N. Armstrong, Tim Shutt, Naweed Syed

**Affiliations:** Hotchkiss Brain Institute and the Alberta Children’s Hospital Research Institute, Cumming School of Medicine, University of Calgary, Calgary, Alberta T2N 2T9 Canada; The Department of Anesthesiology, Cumming School of Medicine, University of Calgary, Calgary, Alberta T2N 2T9 Canada; The Departments of Medical Genetics and Biochemistry & Molecular Biology, Cumming School of Medicine, University of Calgary, Calgary, Alberta T2N 2T9 Canada; The Department of Biology, College of Arts and Sciences, Saint Louis University, Saint Louis, MO 63103-2010 USA; The Department of Cell Biology & Anatomy and the Alberta Children’s Hospital Research Institute, Cumming School of Medicine, University of Calgary, Calgary, Alberta T2N 4Z6 Canada

**Keywords:** General anesthetics, Neurotoxicity, Sevoflurane, Desflurane, Mitochondria, Synapse

## Abstract

**Background:**

Concerns have risen regarding the potential side effects of clinical exposure of the pediatric population to inhalational anesthetics, and how they might impact cognitive, learning, and memory functions. However, neither the mechanisms of anesthetic cytotoxicity, nor potential protective strategies, have yet been fully explored. In this study, we examined whether two of the most commonly used inhalational anesthetics, sevoflurane and desflurane, affect neuronal viability and synaptic network assembly between cultured rat cortical neurons.

**Results:**

Primary rat cortical neuron cultures were exposed to equipotent sevoflurane or desflurane for 1 hour. Neuron viability, synaptic protein expression, mitochondrial morphology, and neurite growth were assayed with immunostaining and confocal microscopy techniques. The effects of anesthetics on the functional development of neural networks were evaluated with whole-cell patch clamp recordings of spontaneous synaptic currents. Our results demonstrate that an acute exposure to sevoflurane and desflurane inhibits the development of neurite processes, impacts the mitochondria, and compromises synaptic proteins - concomitant with a reduction in synaptic function in mature networks. Interestingly, pretreatment of neurons with a mitochondrial division inhibitor (Mdivi-1) not only protected mitochondria integrity but also played a protective role against anesthetic-induced structural and functional neurotoxicity.

**Conclusions:**

We show that Mdivi-1 likely plays a protective role against certain harmful effects of general anesthetics on primary rat neuronal cultures. In addition, Mdivi-1 alone plays a direct role in enhancing growth and modulating synaptic activity. This study highlights the importance of further study into possible protective agents against anesthetic neurotoxicity.

## Background

General anesthetics are required for surgical procedures. Whereas our understanding of various modes of anesthetic actions has improved significantly [1], their use in younger children has brought to light significant long-term side effects as they pertain to compromised learning and memory functions [2]. These agents act mainly as agonists of gamma-aminobutyric acid (GABA) receptors, antagonists of the N-methyl-D-aspartate (NMDA) receptors, or modulators of the fluidity of plasma membrane [3].

The potential neurotoxic effects of anesthesia were first brought to light with the observation that blockade of the NMDA-receptor induces apoptotic neurodegeneration of the developing brain [4]. Extensive epidemiological studies have since questioned the safety of general anesthesia in young children whose brains are undergoing rapid development [2]. A clear consensus in the field vis-à-vis the potential target sites for anesthetic toxicity have yet to be reached-due mainly to myriad confounding variables. Whereas it is becoming increasingly clear that either single or repeated exposure of children to anesthetics impacts their cognitive functions including learning and memory, the mechanisms remain poorly defined. Since learning and memory rely on synaptic integrity and plasticity [5], it stands to reason that inhalational anesthetics affect synaptic assembly in children, however this cannot be tested experimentally. Animal models involving either in vivo or in vitro techniques have now shown that general anesthetics induce neuroapoptosis, leading to neurodevelopmental delays or cognitive disabilities in later life [6]. For instance, administration of propofol to neonatal rats [7], and rhesus macaques [8] increased cell death in the cortex and hippocampus, which led to long-term learning and memory deficits. Similar results have emerged whereby other general anesthetics were shown to impact neurogenesis, proliferation, and differentiation [9, 10]. However, the data demonstrating the acute effects of anesthetic exposure to synaptic architecture and function in developing networks is still lacking-specifically for sevoflurane and desflurane.

In this study, we hypothesized that exposure of immature neurons to clinically relevant doses of sevoflurane and desflurane will negatively impact neuronal viability and synaptic function, and that these effects may involve compromised synaptic proteins and mitochondrial integrity. To test this hypothesis, we used cultured rat cortical neurons, which were exposed to sevoflurane and desflurane, and their cytotoxic effects were investigated. We next sought strategies that might curtail anesthetic-mediated cytotoxicity in an in vitro cortical neuron model.

## Results

### Desflurane and sevoflurane inhibited neurite outgrowth and reduced cell survival in immature rat cortical neurons

We exposed rat cortical neurons to equipotent, physiologically relevant doses of sevoflurane and desflurane. Specifically, cortical cultures were exposed to clinically relevant concentrations (roughly 0.5 minimum alveolar concentration equivalent) of either sevoflurane (1.3 %) or desflurane (4.3 %) [11, 12].

**Fig. 1:**
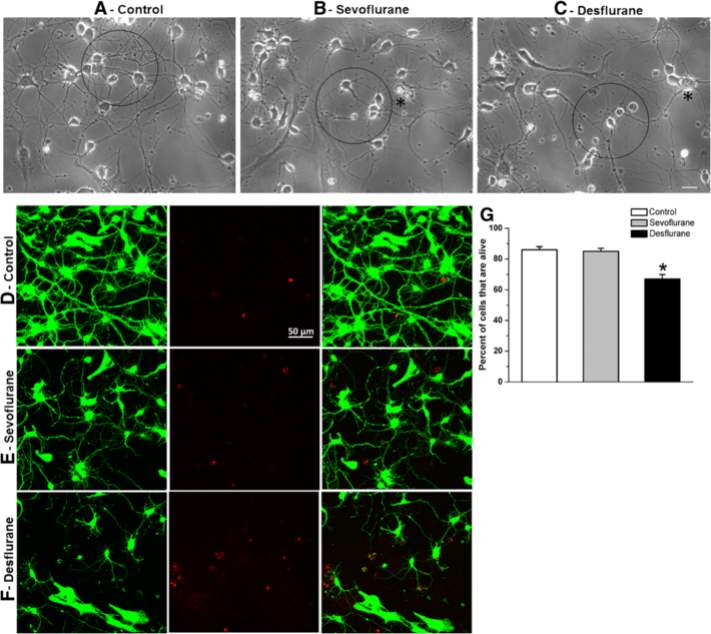
Sevoflurane and desflurane inhibited neurite outgrowth and compromised neuronal viability of rat cortical neurons. (**a–c**) Phase contrast images taken at day 3 of random areas of neurons exposed to medical air only (**a**), or equipotent sevoflurane (**b**, 1.3 %) and desflurane (**c**, 4.3 %). Circled areas indicate neurons and neurite overlap, and asterisks indicate neurons with the vacuolation typical of apoptosis. (**d–f**) Confocal images were taken on day 3 stained for live cells with Calcein-AM (green) and ethidium homodimer (red) for dead cells. (**g**) Quantification of live/dead cells by counting all cells positive for Calcein-AM or ethidium homodimer staining within at least three given areas of at least four replicate dishes. Scale bar 25 μm (**a–c**), 50 μm (**d–f**).

To qualitatively examine how anesthetics may impact neurite growth and viability, neonatal neurons were exposed for 1 h to medical air (alone, control) or medical air mixed with sevoflurane or desflurane and maintained in culture for 3 days. Phase contrast images were acquired on day 3 to evaluate early neuronal morphology. Figure 1a shows a culture dish grown under control conditions (medical air only), yielding a healthy population of cells with extensive neurite branches, processes, and overlapping neurites (indicated by a circle). Although cells exposed to sevoflurane (Fig. 1b) and desflurane (Fig. 1c) developed neurites, their total length and number of branches appeared reduced (Fig. 1a). Note that certain cell bodies of sevoflurane and desflurane treated neurons appeared to exhibit vacuolation, indicative of neuronal degeneration and apoptosis (indicated by asterisks, Fig. 1b, c).

To validate increases in the incidence of cell death, we next performed a live/dead cell assay. Specifically, control and anesthetic treated cells were maintained in culture for 3 days. Neurons were incubated and stained with green-fluorescent calcein-AM for 15 mins to indicate intracellular esterase activity (live cells), and red-fluorescent ethidium homodimer-1 to determine damage to plasma membrane integrity (dead cells) (Fig. 1d–f). To quantify these effects, neurons were counted based on a random selection of minimum of three areas (88,090 μm^2^) from each dish for at least four replicate dishes. The mean value of cell viability reflected by the percentage of viable cells (calcein-AM positive staining) was calculated. Cells exposed to sevoflurane (85 ± 2 % alive, *n* = 17 areas, *p* > 0.05) did not show a difference (*p* = 0.84) in cell viability compared to control cells (86 ± 2 % alive, *n* = 16 areas), whereas those subjected to an equipotent dose of desflurane (67 ± 3 % alive, *n* = 14 areas) revealed a significant reduction in cell viability (*p* < 0.0001) (Fig. 1g).

### Both sevoflurane and desflurane perturbed the expression of synaptic proteins

**Fig. 2:**
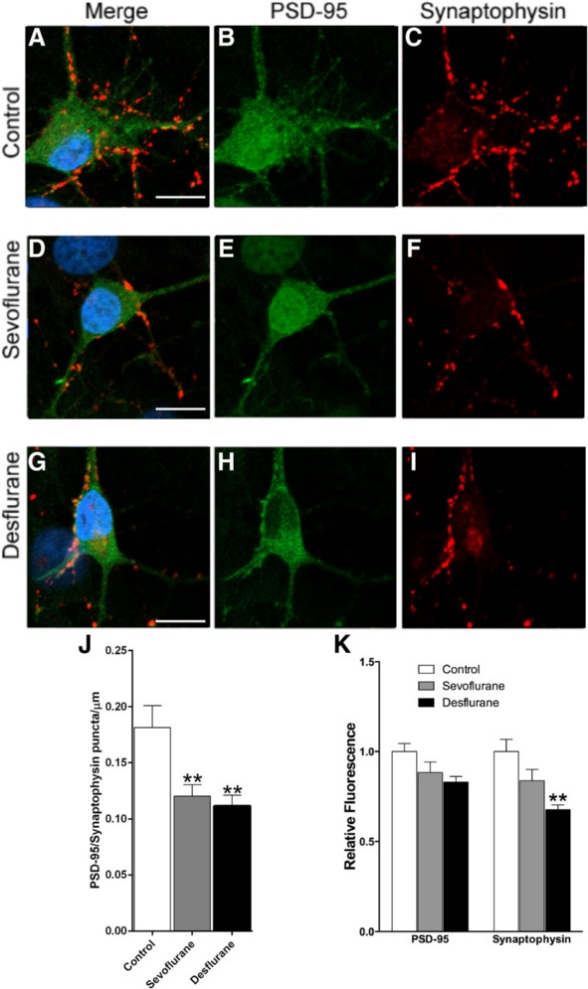
Sevoflurane and desflurane reduced co-localization of pre- and postsynaptic proteins. (**a–i**) Confocal images stained for postsynaptic PSD-95 (green) and presynaptic synaptophysin (red) in neurons 10 days post-culture exposed to sevoflurane and desflurane. (**j**) Quantification of number of PSD-95/synaptophysin positive puncta per μm of neurite. (**k**) Quantification of the mean fluorescence intensity of neurons stained for either PSD-95 or synaptophysin, normalized to the value in the control dish. One-way ANOVA: * *p* < 0.05, ** *p* < 0.01 as determined by pairwise comparison of the post-hoc tests. No asterisk indicates no statistical significance (*p* > 0.05). Scale bar 10 μm

Whereas cell survival and membrane integrity are fundamental to the development of functional neural networks, cell-cell or neurite-neurite contacts form the basis for the development and maturation of synaptic structures. These contacts are maintained by a variety of proteins. To assess whether inhalational anesthetics affect the level and/or localization of two critical synaptic proteins, such as the presynaptic vesicle protein, synaptophysin, and the postsynaptic density protein, PSD-95 [13], day 0 cells were exposed to medical air only (serving as a control, Fig. 2a–c), medical air mixed with sevoflurane (Fig. 2d–f) and desflurane (Fig. 2g–i) for 1 hour. Neurons were maintained in culture for 10 days to allow for the full development of a mature synaptic network, a commonly used time point in primary culture [14].

Quantitative, immunochemical analysis demonstrated that the controls exposed to the medical air alone developed intricate synaptic circuitries represented by a high degree of PSD-95 expression and punctate expression of presynaptic synaptophysin, as well as intense staining for both proteins (Fig. 2a–c). Treatment with sevoflurane and desflurane appeared to have reduced the incidence of punctate expression of synaptophysin, marking a potential decrease in the number of synapses between these neurons (Fig. 2d, g). To quantify this, we counted the number of PSD-95/synaptophysin positive puncta per length of the selected neurite under each condition. Neurites were a heterogenous mix of primary and secondary branches identified as filamentous projections from either a neuron’s cell body or another neurite, respectively. Multiple neurites were analyzed from each cell and at least six cells were analyzed in each condition. These neurites ranged in length from 16.8 μm to 97.7 μm. Indeed, we observed a significant reduction in the number of puncta per μm of neurite as a result of anesthetic treatment (ANOVA F_2,49_ = 7.015, *p* = 0.0021) (Fig. 2j) in both sevoflurane (0.12 ± 0.01 puncta/μm, *n* = 20 neurites, *p* < 0.01) and desflurane (0.11 ± 0.01 puncta/μm, *n* = 14 neurites, *p* < 0.01) as compared to control neurons (0.18 ± 0.02 puncta/μm, *n* = 18 neurites). Furthermore, we measured the mean fluorescence intensity of synaptophysin and PSD-95 in separate images taken with similar cell densities (800–1000 cells/mm^2^) under control and anesthetic-treated conditions. Our data demonstrated that the fluorescence intensity (Fig. 2k), relative to the control, of presynaptic synaptophysin was significantly reduced by anesthetic treatment after analyzing with ANOVA (F_2,28_ = 10.10, *p* = 0.005). However, post-hoc tests revealed a significant difference only with desflurane treatment (0.68 ± 0.03, *p* = 0.0014, n = 6), yet not with sevoflurane (0.84 ± 0.06, *n* = 7, *p* = 0.11). PSD-95 was not significantly reduced by either desflurane (0.83 ± 0.03, *p* = 0.1, n = 6) or sevoflurane (0.88 ± 0.06, *p* = 0.35, n = 7).

### Sevoflurane and desflurane impacted neuronal mitochondria and these effects were inhibited by Mdivi-1

**Fig. 3:**
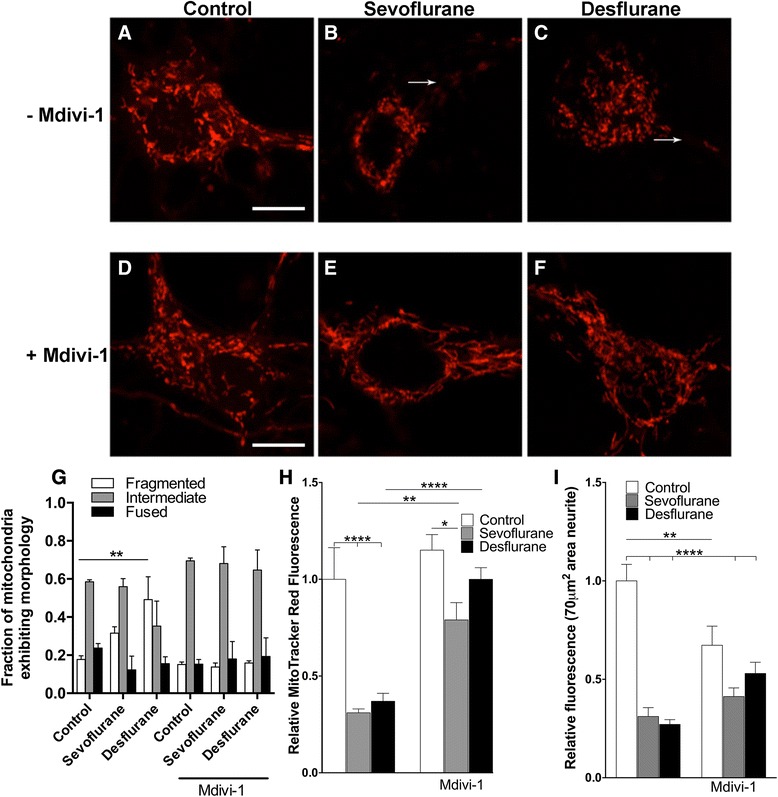
Sevoflurane and desflurane impaired mitochondrial morphology and function, which was rescued by a mitochondrial division inhibitor, Mdivi-1. (**a-f**) Confocal images of individual neurons stained with MitoTracker Red after exposure to the indicated anesthetics in the presence or absence of Mdivi-1 (10 μM) at 6 days post-culture. Arrows indicate neurites, wherever unclear. (**g**) Quantification of predominant mitochondrial morphology in each treatment condition. (**h**) Quantification of mean fluorescence intensity of MitoTracker Red. (**i**) Quantification of mean fluorescence intensity of MitoTracker Red in neurite as measured in 70 μm^2^ regions of interest. Two-way ANOVA. * *p* < 0.05, ** *p* < 0.01, *** *p* < 0.001, **** *p <* 0.0001 as determined by pairwise comparison of the post-hoc tests. Scale bar 10 μm (**a–f**)

All aspects of neuronal growth and synaptic function rely on energy derived from mitochondria. Therefore, we examined mitochondrial morphology with MitoTracker Red, a specific mitochondrial marker with a mitochondrial membrane potential dependent uptake, and whether pre-treatment with a mitochondrial protective agent, Mdivi-1, can help restore any deficits. As shown in Fig. 3a, mitochondria in control neurons exhibit typical rounded rod shapes (intermediate). However, in sevoflurane and desflurane treated neurons, mitochondria appeared fragmented within the cell body, and were scarcely present in the neurites (indicated by arrows) (Fig. 3b, c). Some cells exhibited fused mitochondrial morphology (Fig. 3e). Two-way ANOVA showed a significant interaction (F_10,36_ = 4.171, *p* = 0.0007) between anesthetic treatment and Mdivi-1 pre-treatment, indicating that these two conditions play an interdependent role in mitochondrial morphology. Quantification of mitochondrial morphology (Fig. 3g) showed a robust increase in fragmented mitochondria in desflurane (49.2 % ± 12.0 %, *p* = 0.0098, *n* = 38 cells), as compared to the control (17.7 % ± 2.0 % *n* = 61 cells). Sevoflurane increased the percentage of cells exhibiting fragmented mitochondria (31.6 % ± 3.3 % *n* = 66 cells), but this difference was not significant. Our data also revealed that fluorescence intensity – a measure of mitochondrial membrane potential – in separate whole images with similar densities was significantly reduced as analyzed by a two-way ANOVA with a significant interaction between Mdivi-1 pre-treatment and anesthetic treatment (F_2,33_ = 4.421, *p* = 0.0199), which indicates that Mdivi-1 pre-treatment effects depend upon whether the cells were exposed to either sevoflurane, desflurane, or no anesthetic. This significant reduction was shown in both sevoflurane and desflurane treated neurons (Fig. 3h). Specifically, relative to the fluorescence in the control, desflurane (0.37 ± 0.04, *n* = 10) and sevoflurane (0.31 ± 0.02, *n* = 9) reduced mean fluorescence significantly (*p* < 0.0001 for both). Since there appeared a noticeable reduction in mitochondrial staining within the neurites, a 70 μm^2^ region of interest located at the neurite connection to the cell body was selected and the fluorescence intensity was measured within this region of interest (Fig. 3i). Accordingly, as before, analysis by a two-way ANOVA showed a significant interaction between Mdivi-1 pre-treatment and anesthetic treatment (F_2,63_ = 10.21, *p* = 0.0001). Specifically, desflurane treated neurites exhibited reduced relative fluorescence staining (0.27 ± 0.024, *n* = 13 neurites *p* < 0.0001), as did sevoflurane treated neurons (0.31 ± 0.044, *n* = 13 neurites *p* < 0.0001) when compared to control neurites.

The above experiments clearly demonstrated that sevoflurane and desflurane induced mitochondria fragmentation and reduced fluorescence intensity. We next examined whether pharmacological inhibition of mitochondria division by a mitochondrial division inhibitor, Mdivi-1, could reduce anesthetic-induced changes in their morphology. Mdivi-1 was chosen since it is known to protect neuronal cell death, and it inhibits mitochondrial fragmentation [15]. To this end, neurons isolated from rat pups were first pre-treated with Mdivi-1 (10 μM [16], approximately the IC_50_ for this compound) for 1 h before anesthetic exposure. MitoTracker Red staining and confocal microscopy studies showed that Mdivi-1 pretreatment prevented anesthetic induced changes in mitochondrial morphology (Fig. 3e–g) as well as fluorescent intensity (Fig. 3h, i). Figure 3e, f clearly show that mitochondria in neurons exposed to sevoflurane and desflurane still displayed rod shapes which were distinct from the fragmented morphology seen in neurons without Mdivi-1 pretreatment (Fig. 3b, c). Additionally, quantification of mitochondrial morphology (Fig. 3g) showed that Mdivi-1 pre-treatment restored mitochondrial morphology for sevoflurane and desflurane treated cells to values near control. Specifically, the percentage of cells exhibiting fragmented mitochondria was 15.1 % ± 1.3 % (*n* = 39 cells) in control, 13.8 % ± 2.1 % (*n* = 46 cells) in sevoflurane, and 15.9 % ± 1.2 % in desflurane (*n* = 42 cells) treated cells (Fig. 3h). Similarly, other analyses revealed that Mdivi-1 treatment increased the relative fluorescence values as compared to similar anesthetic treatment without Mdivi-1 pre-treatment (Fig. 3h) for desflurane (1.01 ± 0.06, *n* = 4, *p* < 0.0001) and for sevoflurane (0.79 ± 0.09, *n* = 5, *p* = 0.0018). Furthermore, the reduction in neurite fluorescence intensity by anesthetics was also altered by pretreatment with Mdivi-1. Specifically, the neurites of Mdivi-1 treated cells that were also exposed to desflurane (0.53 ± 0.057, *n* = 10 neurites) and sevoflurane (0.046 ± 0.13, *n* = 8 neurites) were not significantly lower than Mdivi-1 treated controls cells (Fig. 3i). However, it is important to note that the reduction in the MitoTracker fluorescence in neurites of Mdivi-1 pre-treated control and anesthetic treated cells appears to be a result of the reduction in the control fluorescence (as revealed by ANOVA post hoc analysis) as compared to Mdivi-1 control neurites (*p* = 0.0068). In addition, Mdivi-1 did not significantly increase the fluorescence within the neurites for either sevoflurane or desflurane (*p* > 0.05) when compared to sevoflurane or desflurane alone. These data indicate that Mdivi-1 impacted neurite fluorescence intensity in a way that did not restore these values to Mdivi-1 untreated controls, as it did over the whole image fluorescence.

### Mdivi-1 attenuated sevoflurane and desflurane-mediated changes in neurite growth

**Fig. 4:**
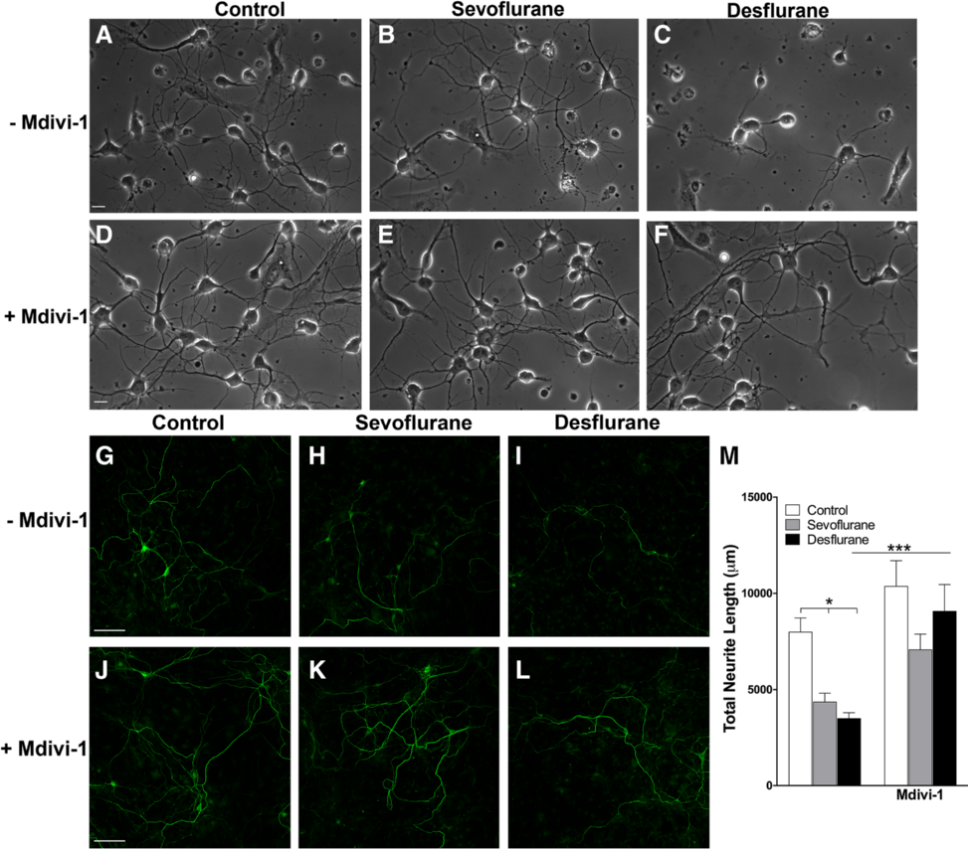
Sevoflurane and desflurane inhibit neurite growth, which is restored with Mdivi-1 pre-treatment. (**a–f**) Phase contrast images of neurons after exposure to the indicated anesthetics in the presence or absence of Mdivi-1 at 3 days post-culture. (**g–l**) Confocal microscopy images of areas stained with 160 kDa neurofilament polyclonal antibodies. (**m**) Quantification of neurofilament staining with automated neurite tracing software, NeuriteTracer. Two-way ANOVA. * *p* < 0.05, ** *p* < 0.01, *** *p* < 0.001 as determined by pairwise comparison of the post-hoc tests. Scale bar 25 μm (**a–f**), 100 μm (**g–l**)

If anesthetic-induced neurotoxicity were directly related to impairment of mitochondrial integrity and function, then protecting the mitochondria should attenuate anesthetic-induced alterations. To test this possibility, cells were pre-treated either with or without Mdivi-1 (10 μM) for 1 h prior to anesthetic exposure. Phase contrast images taken on day 3 post-culture demonstrated that sevoflurane (Fig. 4b) and desflurane (Fig. 4c) compromised growth as compared to control (Fig. 4a); these morphological alterations were absent in Mdivi-1-pretreated neurons exposed to either sevoflurane (Fig. 4e) or desflurane (Fig. 4f). To confirm these observations in the phase contrast images, we stained cells with polyclonal antibodies against the 160 kDa fragment of neurofilament (Fig. 4g–l). Reductions in the total extent of neurite growth were qualitatively noted. We then analyzed these images (minimum *n* = 10 areas) using automated neurite tracing software (NeuriteTracer [17], Fig. 4m). Areas of 0.41 mm^2^ were imaged and the total neurite length based on identical threshold values for each condition was measured. We noted a dramatic reduction when analyzed by a two-way ANOVA for the anesthetic treatment (main effect F_2,61_ = 8.879, *p* = 0.0004), growth was then enhanced by Mdivi-1 pre-treatment alone (main effect F_1,61_ = 24.37, *p* < 0.0001). There was a non-significant Mdivi-1 pre-treatment and anesthetic treatment interaction (F_2,61_ = 1.970, *p* = 0.1482). Specifically, the total neurite length in sevoflurane was 4354 ± 461 μm (*p* = 0.029) and in desflurane was 3479 ± 305 μm (*p* = 0.012), which were significantly reduced as compared to that in control images (7984 ± 738 μm). In Mdivi-1 pre-treatment conditions these values were near control values (10371 ± 1331 μm) for both desflurane (9067 ± 1395 μm) and sevoflurane (7066 ± 814 μm) (Fig. 4m).

### Sevoflurane and desflurane impaired functional synaptic development, which is rescued by Mdivi-1 pre-treatment

**Fig. 5:**
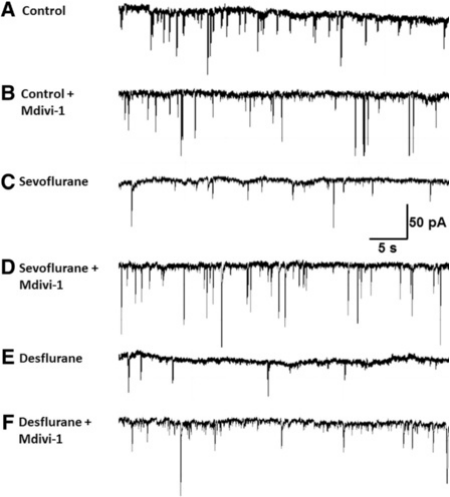
Sevoflurane and desflurane reduced spontaneous synaptic events and these effects were rescued by pre-treatment with Mdivi-1. (**a–f**) Representative patch clamping recording traces of spontaneous synaptic currents in neurons exposed to no anesthetic (control), desflurane, and sevoflurane alone or with Mdivi-1 (10 μM) pre-treatment, as indicated, recorded at 10 days post-culture. Spontaneous synaptic currents were isolated by adding tetrodotoxin (0.5 μM) in external solution

**Fig. 6:**
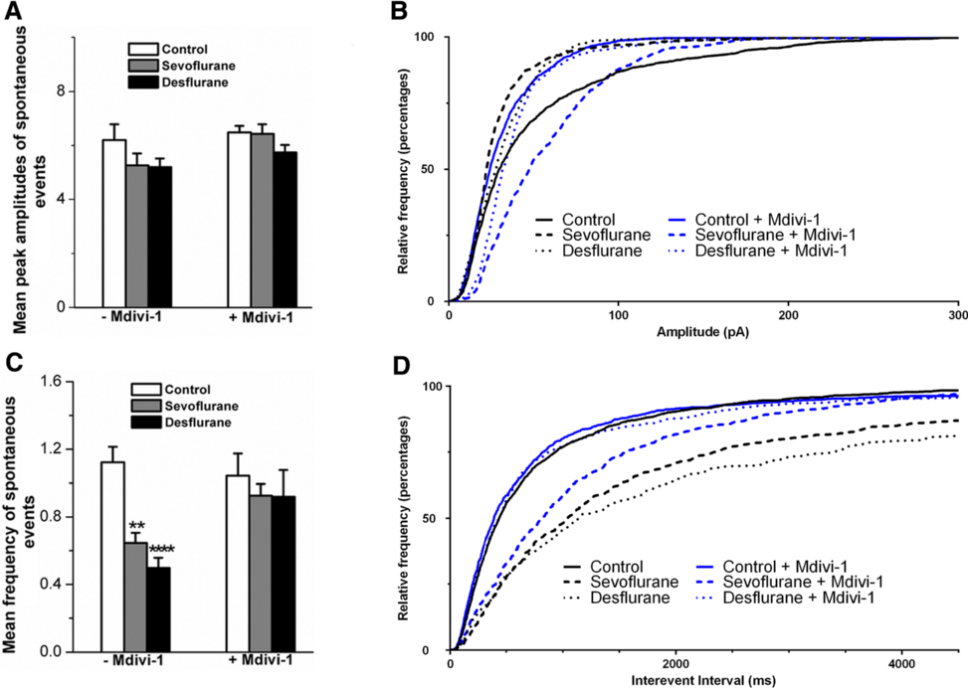
Statistical data depicting the effects of sevoflurane and desflurane with and without Mdivi-1 pretreatment on the amplitude and frequency of spontaneous synaptic events. The mean spontaneous current amplitude was quantified in (**a**), and a cumulative probability plot of the event amplitude (pA) of all events recorded (minimum 400 events) in (**b**). Quantification of mean spontaneous current frequency (**c**) and a cumulative probability plot of the interevent interval (ms) of all events recorded (minimum 400 events) (**d**). Two-way ANOVA. * *p* < 0.05, ** *p* < 0.01, *** *p* < 0.001, **** *p* < 0.0001 as determined by pairwise comparison of the post-hoc tests; cumulative probability plot analyzed with Kolmogorov-Smirnov test

We have shown that sevoflurane and desflurane caused cellular damage by affecting synaptic machinery and mitochondria. We next asked if these structural alterations could lead to functional network abnormalities, and if pretreatment with Mdivi-1 might improve the development of a neuronal network. Specifically, we tested whether anesthetics affect spontaneous synaptic current development in pyramidal neurons, which are implicated in cognitive functions. To this end, a pyramidal neuron was identified based on its distinct morphological characteristics in culture, namely a triangular shaped cell body with a prominent apical dendritic process and several shorter basal dendrites [18]. Whole cell voltage-clamp experiments were performed on neurons with and without pretreatment with Mdivi-1 at 10–14 days post-culture. On each recording day, one dish from each condition was recorded to balance possible time-dependent changes. Spontaneous inward currents were isolated in TTX-containing external solution at a holding potential at −70 mV. Sample traces of control, sevoflurane and desflurane with and without Mdivi-1 are illustrated in Fig. 5, and the quantification of synaptic current frequency and amplitude data is shown in Fig. 6. The mean peak amplitudes of synaptic currents were not significantly changed as analyzed by two-way ANOVA for either Mdivi-1 pre-treatment (main effect F_1,65_ = 7.232, *p* = 0.0694), or sevoflurane or desflurane treatment (main effect F_2,65_ = 2.002, *p* = 0.1433). In addition, the Mdivi-1 pre-treatment and anesthetic treatment interaction F_2,65_ = 1.109, *p* = 0.5954 was not significant (Fig. 6a). Specifically, the mean peak amplitudes in non-Midi-1 treated neurons were 6.2 ± 0.58 (control, n = 15), sevoflurane (5.27 ± 0.43, *n* = 12, *p* > 0.05), desflurane (5.2 ± 0.32, *n* = 14, *p* > 0.05), and in Mdivi-1-treated group were 6.49 ± 0.24 (control, *n* = 13), sevoflurane (6.43 ± 0.35, *n* = 9, *p* > 0.05) and desflurane (5.74 ± 0.28, *n* = 7, *p* > 0.05). To further test whether anesthetic treatment may affect the amplitude distribution of spontaneous synaptic currents (despite having similar means) these data were further analyzed using cumulative probability functions as shown in Fig. 6b. Indeed, the data shows that each condition has a slightly different amplitude distribution. In fact, when analyzed with the Kolmogorov-Smirnov test each condition’s cumulative probability function was significantly different: sevoflurane compared to control + Mdivi-1 was *p* = 0.0040 and all others were *p* < 0.0001. Since the Kolmogorov-Smirnov test tests whether the median, variance, or shape of cumulative probability functions are different, these results indicate that although the mean amplitudes did not change (Fig. 6a), the distribution of all amplitude events was slightly different among different treatment conditions. All traces, except for sevoflurane + Mdivi-1 and desflurane + Mdivi-1, were entirely shifted to the left of the control trace, indicating that there was a somewhat larger probability of smaller amplitude, spontaneous synaptic currents in these conditions. Interestingly, both sevoflurane and desflurane with Mdivi-1 pre-treatment was shifted to the right of the control trace for low amplitude events, indicating these conditions had a reduced frequency of low amplitude events.

The mean frequency of spontaneous events was evidently altered by both sevoflurane and desflurane (Fig. 6c, d). The spontaneous inward currents in control pyramidal neurons (Fig. 5a) were robust, indicating the development of a functional network, while both sevoflurane and desflurane (Fig. 5c–e; Fig. 6c, d) reduced the frequency of these synaptic currents. Interestingly, the reduction in the frequency of synaptic events was rescued by pretreatment of neurons with Mdivi-1 (Fig. 5d–f; Fig. 6c, d). This was analyzed with a two-way ANOVA, which showed a significant Mdivi-1 pre-treatment and anesthetic treatment interaction (F_2,64_ = 3.286, *p* = 0.0438), indicating again that Mdivi-1 pre-treatment effects depend whether the cells were exposed to anesthetic or not. Specifically, the mean synaptic frequency in control neurons was 1.12 ± 0.09Hz (*n* = 15), which was reduced to 0.65 ± 0.06Hz (*n* = 12, *p* = 0.0081) by sevoflurane, and to 0.5 ± 0.06Hz (*n* = 14, *p* < 0.0001) by desflurane (Fig. 6c). In the Mdivi-1 pre-treatment group, the mean frequency in control neurons was 1.04 ± 0.13Hz (*n* = 13), 0.93 ± 0.07Hz (*n* = 9, *p* > 0.05) in sevoflurane treated neurons and 0.92 ± 0.16Hz (*n* = 7, *p* > 0.05) in desflurane exposed neurons (Fig. 6c), respectively. To further evaluate the effects of Mdivi-1 on the distribution of spontaneous synaptic currents, we plotted the interevent interval of each current in a cumulative probability plot (Fig. 6d). We then analyzed these cumulative probability plots for statistical significance with the Kolmogorov-Smirnov test. Sevoflurane (*p* < 0.0001), and desflurane (*p* < 0.0001) cumulative probability functions were dramatically shifted to the right, as compared to the control trace, which indicates there was a dramatic increase in the interevent interval in both anesthetic treated conditions. Furthermore, Mdivi-1 pre-treatment shifted the cumulative probability of the interevent interval to the left, as compared to non-Mdivi-1 treated cells with similar anesthetic exposure, for both sevoflurane (*p* = 0.008) and desflurane (*p* < 0.0001), indicating a restoration towards a distribution comprising more frequent short interevent intervals, as seen in control. However, Mdivi-1 pre-treatment of sevoflurane resulted in a cumulative probability function that was still significantly different than that of the control (*p* < 0.0001) – indicating that Mdivi-1 pre-treatment resulted in a distribution that significantly differed not in mean (Fig. 6c), but rather the shape of the probability function was not fully restored to that of control. Meanwhile, Mdivi-1 pre-treated control traces were not different than non-treated control traces (*p* = 0.0762).

## Discussion

This study provides the first integrative evidence on the molecular and functional effects of sevoflurane and desflurane on the developing neural circuitry in rats. We have also provided the first direct evidence that Mdivi-1, a mitochondrial protective agent, may hold potential to improve some, but not all, of the negative effects of desflurane and sevoflurane on neurons in culture.

In recent years, concerns have been raised regarding neurotoxic side effects of anesthetic exposure in young children; these effects have since been linked to neurocognitive and behavioral deficits [2, 6]. Limited data however exist for the effects of anesthetics on the molecules and mechanisms that underlie synapse formation and plasticity. This information is critical because developing neurons and their synaptic connectivity is in turn contingent upon neuronal firing patterns during a critical period; the silencing of which is the prime target of anesthetic actions. Perturbation of neuronal activity, even over a short time period, is likely to have a long-term impact on neuronal connectivity, which forms the basis for cognitive functions In this study, we provided an account of the effects of two extensively used volatile anesthetic agents and found sevoflurane and desflurane to compromise the development of functional synapses. Specifically desflurane, but not sevoflurane, significantly reduced the level of the critical presynaptic protein synaptophysin. Concurrently, both sevoflurane and desflurane reduced the density of synaptic protein puncta along neurites. As a result, both sevoflurane and desflurane reduced the frequency of spontaneous synaptic currents. Previous studies in animal models have revealed that the loss of synaptophysin or PSD-95 in the cortex results in cognitive decline and behavioral deficits [19–21]. Indeed, exposure to 2.5 % sevoflurane in mice during the early stages of neuronal development results in learning and memory deficits that are accompanied by reduced PSD-95 protein expression in the hippocampus [22]. These data are consistent with our observations in developing rat cortical neurons. Together, these data provide preliminary insights into a potential mechanism involving synaptic proteins by which anesthetics may lead to long-term cognitive impairments. However, our study highlights that the effects of general anesthetics go beyond those that can be explained by transient disruptions in neural activity and communication, since our cultures were exposed to anesthetic prior to the formation of synapses.

Interestingly, the cellular targets of general anesthetics are yet to be fully understood, which may underlie their neurotoxicity. It is known that the mechanism of action of general anesthesia involves targeting ligand-gated ion channels (e.g. receptors of NMDA, GABA_A_, nACh, serotonin etc.), voltage-gated ion channels (e.g. sodium, potassium, calcium etc.), and background channels (e.g. two pore domain potassium channels) to induce anesthesia [3, 23–25]. Although the activities of these receptors and ion channels play important roles in neurite outgrowth, synaptic transmission, and plasticity, it is unlikely that anesthetic targeting of only one of these factors could suffice to account for all aspects of their clinical toxicity. In addition, the distribution and localization of these channels in different neurons and brain regions are widely diverse and the effects of anesthetics may often be transient and reversible upon anesthetic removal. It is therefore unlikely that anesthetic neurotoxicity involves these channels only. Neurite outgrowth, synaptic transmission, and plasticity rely critically on intracellular energy sources. Therefore, it stands to reason that shutting down mitochondrial function may impair all of the above processes, creating a cascade of events, which could lead to neurodegeneration. Mitochondria dysfunction has been implicated in a variety of neurodevelopmental and genetic disorders [26]. Mitochondrial dynamics include fission and fusion, and mitochondrial fission has been closely linked to apoptotic pathway [27]. Indeed, emerging evidence indicates that anesthetic neurotoxicity may involve changes in internal organelles including the mitochondria [28, 29]. For instance, general anesthetics (isoflurane, nitrous oxide, and midazolam) alone, or in combination, cause chronic impairment of mitochondria morphogenesis and synaptic transmission in the developing rat brain [30]. Consistent with these studies, our data clearly demonstrate that both desflurane and sevoflurane trigger mitochondria fragmentation leading to impairment of functional synapse development. Interestingly, we also demonstrated that pre-treatment of in vitro cortical neuron cultures with Mdivi-1 prior to anesthetic exposure significantly attenuated their effects on neural growth (Fig. 4), synaptic currents (Figs. 5 and 6), as well as mitochondrial integrity (Fig. 3).

Mdivi-1 is a highly efficient and selective inhibitor of the mitochondrial division protein Drp1. It inhibits mitochondrial division by binding to Drp1, preventing a conformational change that allows Drp1 self-assembly and GTP hydrolysis, resulting in the formation of “netlike” mitochondria [16]. It has been further shown that Mdivi-1 reduces apoptosis by inhibiting mitochondrial outer membrane permeability in vivo and blocks Cytochrome *c* release in vitro [16]. Recent studies have proven the neuroprotective effects of Mdivi-1 against glutamate excitotoxicity and oxygen-glucose deprivation in vitro and ischemic brain injury in vivo [31]. Mdivi-1 has also been found to protect against doxorubicin-induced cardiotoxicity [32] and pilocarpine-induced seizures in rat hippocampal neurons [33]. In this study, we revealed that pre-treatment with Mdivi-1 effectively inhibits sevoflurane and desflurane-triggered cell damage and synapse attenuation. There exists other evidence supporting potential neuroprotective strategies against anesthesia-induced toxicity in the developing brain. For instance, Pramipexole, a drug known to protect mitochondrial integrity, was found to prevent cognitive decline from early anesthesia exposure in rats [34]. Antioxidant agents such as lPPX and EUK-134 have also been demonstrated to prevent anesthetic-induced changes in mitochondrial integrity and cognitive dysfunction [34]. Taken together, it would appear that strategies targeting mitochondrial structure and function might offer a therapeutic approach against anesthetic-induced neuronal cytotoxicity.

## Conclusions

In summary, our data support the notion that inhalational anesthetics perturb developing neural circuits during critical periods of synaptogenesis. This perturbation of synaptic architecture and function may partly contribute to the behavioral deficits observed in neonatal rat or mouse models after exposure to anesthetics [35, 36]. Our study, together with others, underscores the importance of cautious use of desflurane and sevoflurane in clinical settings – especially in infants and pregnant women when the central nervous system of babies is undergoing rapid development. In addition, the data presented here suggest that mitochondria are likely one of the main cellular targets for anesthetic neurotoxicity. Future strategies should involve exploring various protective roles of pharmacological agents, such as Mdivi-1, at both the cellular, and behavioral levels.

## Methods

### Rat cortical neuronal culture and anesthetic exposure

All animal procedures were in accordance with the standards established by the Canadian Council on Animal Care and with the University of Calgary Animal Care and Use Policy.

Sprague-Dawley rat frontal cortexes were removed from postnatal day 0 pups and enzymatically dissociated with papain (50 u/mL). Cells were then triturated with glass pipettes of decreasing size to create a suspension of single cells. Cells were then diluted to an appropriate density in culture media and plated on culture dishes with a glass coverslip coated with laminin (2 μg/mL) and poly-D-lysine (30 μg/mL). Cells were allowed to settle for one hour prior to the addition of additional 2 mL of culture medium. The culture medium used was Neurobasal medium supplemented with 2 % B27, L-Glutamine (200 mM), 4 % FBS, and penicillin-streptomycin (Invitrogen), which was changed (50 % removed and replaced) every 3–4 days. Culture dishes were allowed to settle for an additional 1–2 hours prior to being placed in an airtight modular incubator chamber (Billups-Rothenberg). Anesthesia-medical air gas mixtures were vaporized using a Datex-Ohmeda Aestiva/5 vaporizer and concentrations were monitored with a GE Healthcare Gas Analyzer. Controls for each exposure group consisted of cells that were incubated in the same manner with medical air only. After 1 h of exposure, the neurons were placed back and maintained in an incubator (37 °C, 5 % CO_2_) until use.

### Cell viability assay

The effects of sevoflurane and desflurane on cell viability were tested in developing neurons at day 3 post-culture to avoid the proliferation of glia seen in later cultures that may mask apoptotic effects in neurons. Both the control and anesthetic-exposed groups were evaluated using the LIVE⁄DEAD® Viability⁄Cytotoxicity Kit (Molecular Probes). Specifically, the cells were exposed to calcein-AM (green, live cells) and ethidium homodimer-1 (red, dead cells) dyes at room temperature for 15 min. These preparations were observed under confocal microscopy (LSM 510 Meta, Zeiss) via a 20X objective at 488 nm (green) and 548 (red) excitation wavelength. Fluorescence images (area size of 88,090 μm^2^) were collected using a long pass filter (505 nm), and band pass filter (560–615 nm) for green and red, respectively. The number of live (green) or dead (red) cells were viewed and counted manually using ImageJ software.

### Immunocytochemistry and confocal microscopy

Immunostaining study of synaptic protein markers and neuronal cytoskeleton was conducted on cells in culture for at least 10 days to allow for the development of a network and maturation of synaptic components [14]. Fixation was performed with 4 % paraformaldehyde and 15 % picric acid fixative solution for 1 h at room temperature. The fixed cultures were permeabilized for 1 h with incubation media consisting of 0.1 % Triton X with 5 % goat serum. A primary rabbit monoclonal antibody for synaptophysin (1:500 dilution) (Abcam) and mouse monoclonal antibody for PSD-95 (1:2000 dilution) (NeuroMab) were applied to the cultures overnight at 4 °C. Secondary antibodies of AlexaFluor 488 goat anti-mouse IgG (1:200 dilution) (Invitrogen) and AlexaFluor 546 goat anti-rabbit (1:200 dilution) (Invitrogen) were then applied. Dishes were mounted with MOWIOL mounting media with DAPI to stain nuclei (Sigma-Aldrich). A similar procedure was followed for the staining of neurofilament, with the exception of the types of primary and secondary antibodies used. A chicken polyclonal antibody against the 160 kDa fragment of neurofilament (1:500 dilution) (Novus Biologicals) was applied to the cultures after permeabilization. The secondary antibody of AlexaFluor 488 goat anti-chicken (1:200 dilution) was applied to the cultures after washing off the primary antibody. Negative control experiments were performed at the same time to test the specificity of synaptophysin, PSD-95, and neurofilament antibodies. No immunofluorescence was detected when primary antibodies were excluded from the staining procedures (data not shown). Image acquisition parameters (laser intensity, pinhole sizes, exposure times, gain settings etc.) were kept the same for both control and anesthetic treated neurons.

To assess the impact of anesthetics on the integrity and function of mitochondria, cortical cultures at 4 days post-culture had their media replaced with warm Hanks’ Balanced Salt Solution with HEPES (10 mM), L-glutamine (2 mM), and succinate (100 μM). MitoTracker Red CMXRos (200 nM) containing HBSS medium was then incubated with the cells for 15 mins at 37 °C. Cells were imaged at this time point to minimize the impact of glia proliferation that would complicate neuron identification. The percentage of cells that exhibited a given mitochondrial morphology (fragmented, intermediate, fused) was counted in triplicate experiments. Each cell was classified as having a given mitochondrial morphology based on the predominant mitochondrial morphology present in each cell according to the following criteria: mitochondria were considered fragmented when less than 0.75 μm in length, intermediate when 0.75 μm to 3 μm in length, and fused when greater than 3 μm in length.

Fluorescence images were taken with a Nikon Eclipse C1Si confocal microscope (Nikon Instruments Inc.). Fluorescent intensity was measured exclusively in separate image sets taken with identical confocal parameters for all treatment conditions. Fluorescence intensity was measured over the entire field of view with similar cell densities (approx. 1000 cells/mm^2^) for each fluorescent dye used. Fluorescence units were standardized (“relative fluorescence ratio”) to control images taken from the same culture that were fixed and stained at identical time points.

### Whole-cell patch clamp recording of synaptic currents

Whole-cell recordings of spontaneous synaptic currents were performed on pyramidal cortical neurons after 10 days (to correlate with synaptic protein staining) in culture using a Multiclamp 700B amplifier connected to an analog-to-digital interface Digidata 1322 (Axon Instruments). Signals were recorded with pClamp 9.2 software (Axon Instruments) under voltage-clamp mode with a holding potential of −70 mV. The external solution contained (in mM) NaCl, 135; CaCl_2_, 3; KCl, 5; MgCl_2_, 2; HEPES, 10; D-Glucose, 10; pH adjusted to 7.3 with NaOH; ~ 330 mOsm, this solution included tetrodotoxin (TTX, 0.5 μM) in order to block action potential-dependent synaptic events. The internal pipette solution was composed of (in mM) CsCl, 140; CaCl_2_, 1; MgCl_2_, 2; HEPES, 10; EGTA, 11, ATP-Mg, 2; GTP-Na, 0.6; pH adjusted to 7.3 with CsOH; ~ 300 mOsm. Borosilicate patch pipettes (A-M Systems, Inc) pulled to a tip resistance of 3–5 MΩ were exclusively used. Only cells with a series resistance less than ~15 MΩ and leak currents less than ~100 pA were included.

### Statistical analysis

Spontaneous events were analyzed with Mini Analysis software (Synaptosoft). Peaks of events were visually screened and selected if they displayed a monophasic rise time to peak, an amplitude larger than the detection threshold (>5 pA) set at the maximum of the background noise. Synaptic events, acquired for 100 s after the initial 1–2 min of recording of basal activity, were used for frequency and amplitude analysis.

Statistical significance test was run using OriginPro 8.0 PRO or GraphPad. One-way ANOVA was used to compare groups with only one factor (ie. anesthetic exposure). Two-way ANOVA was used to compare groups with two factors (ie. anesthetic exposure and Mdivi-1 treatment). Post-hoc tests with Bonferroni test were conducted. A square root transform was applied to the mean frequency and mean peak amplitude of spontaneous events to stabilize changes in variance as a conservative approach to the violation of homogeneity in order to perform ANOVA. Data shown was not transformed. Cumulative synaptic event plots were tested for statistical significance with the Kolmogorov-Smirnov test. Values were graphed as mean ± S.E.M. Differences between data sets were considered significant if appropriate statistical tests resulted in *p* values smaller than 0.05; *p* values were reported for the post-hoc tests, except where indicated otherwise.
